# Prevalence and Factors Associated with Home Childbirth with Unskilled Birth Assistance in Dodoma-Tanzania: A Cross Sectional Study

**DOI:** 10.24248/eahrj.v4i1.626

**Published:** 2020-06-26

**Authors:** Fabiola Vincent Moshi, Glorialoveness Lymo, Nyasiro S. Gibore, Stephen M. Kibusi

**Affiliations:** a Department of Nursing and Midwifery, College of Health Science of University of Dodoma; b Department of Public Health, College of Health Science of University of Dodoma

## Abstract

**Background:**

Improving maternal health is one of the goals to be achieved under the Sustainable Development Goal (SDG) number 3. Worldwide, half a million of women die each year from pregnancy and childbirth related complications which can be prevented by skilled birth assistance. One of the determinants of maternal health is place of childbirth. Giving birth at home leads to a high risk of maternal and child mortality. The aim of the study was to determine the prevalence and factors associated with choice of home childbirth in Dodoma Municipality.

**Methods:**

A community based cross section study using multistage sampling was used to obtain the sample in which 2,523 women who gave birth within 3 years prior to the date of the study from different wards of Dodoma municipal were interviewed. The data obtained were entered and analysed using SPSS version 20. Binary logistic regression analysis was used to establish predictors of home childbirth with unskilled birth assistance.

**Results:**

A total of 1,174 (46.5%) women had home childbirth with unskilled birth assistance. After adjusted for the confounders, predictors of home childbirth with unskilled birth assistance among study respondents were level of education [primary education, AOR=0.69 at 95% CI=0.557-0.854, p<.001; secondary education, AOR=0.492 at 95% CI=0.358-0.676, p<.001 and above secondary education, AOR=0.35 at 95% CI=0.16-0.765;p<.01]; marital status [married women, AOR=0.686 at 95% CI=0.547-0.86, p<.001]; occupation of a mother [peasant, AOR=1.508 at 95% CI=1.214-1.874, p<.05]; parity [2 to 4 children, AOR=1.316 at 95% CI=1.028-1.684, p<.05; More than 4 children, AOR=2.006 at 95% CI=1.427-2.82, p<.001]; number of antenatal visits [4 or more antenatal visits, AOR=0.451 at 95% CI=0.204-0.997, p<.05] and walking distance [less than 5kilometres, AOR= 0.797 at 95% CI=0.674-0.943, p<.001]

**Conclusion:**

The findings of this study suggest a need for health education in the community on the importance of skilled birth delivery. There is also a need for the government to roll out the implementation of Primary Health Services Development Program (PHSDP-MMAM) which addresses the delivery of health services within 5 kilometres to ensure fair, equitable and quality health services to the community.

## BACKGROUND

Maternal mortality rate is still a challenge to public health system worldwide. Globally, in 2015 about 303,000 women died due to pregnancy and childbirth with 99% of this death occurring in developing countries.^1^ Almost all of these deaths occurred among rural poor communities with unskilled birth assistants.^1^ Between 1990 and 2015, maternal mortality declined worldwide from 385 to 216 deaths per 100,000 live births.^2^ However maternal mortality rate remain unacceptably higher in sub Saharan countries with the lifetime risk of dying during pregnancy being 1 in every 41 women.^1^ Tanzania is among the developing countries with highest maternal mortality ratio, estimated at 556 deaths per 100,000 live birth.^3^

Most women in developing countries die due to complications that arise during pregnancy and while giving birth. Maternal deaths are caused by both direct and indirect cause. About 75% of these deaths are caused by direct causes such as severe bleeding, infections, high blood pressure and unsafe abortion while the other 25% being contributed by diseases like malaria and Acquired Immune Deficiency Syndrome (AIDS).^4^

More than 2.5 million neonates died within their first 28 days of life in 2016, contributing up to 45% of global under 5 deaths worldwide.^6^ Globally, the trend of neonatal mortality reportedly decreased from 37 deaths per 1,000 live births in 1990 to 19 deaths per 1,000 live births in 2016.^7^

The burden of neonatal mortalities in sub-Saharan Countries is high, however, the trend of neonatal mortality showed a decrease from 46 neonatal deaths per 1000 live births in 1990 to 26 neonatal deaths per 1000 live births in 2016.^7^ 77% of these deaths occurred in 2 regions, the Southern Asia (39%) and sub-Saharan Africa 38%.^7^

Tanzania is among the countries in Sub-Saharan region with the highest neonatal mortality rate of 25 deaths per 1,000 live births.^4^

There exist variations in neonatal mortalities in Tanzania. Dodoma region located in central Tanzania is among the regions with high neonatal mortality (29 deaths per 1,000 live births) above the national average.^4^

Globally, in the year 2015, infectious diseases, prematurity and complications during labour and delivery were the main causes of deaths among neonates.^4^ Other causes of death were preterm birth complications, sepsis and *meningitis*.^8^ There is direct relationship between low use of skilled health personnel for delivery and maternal and neonatal mortalities. As clearly defined by WHO “skilled health personnel” are competent maternal and newborn health professionals educated, trained and regulated to national and international standards.^1^ Birth attended by skilled health personnel have proven to reduce maternal and neonatal mortalities.^9^ Worldwide up to 80% of birth were attended by a skilled personnel in the latest 2012 to 2017.^1^ Sub Saharan Africa has also shown some advancement over the same period with about 50% of deliveries attended by a skilled personnel.^1^ In the year 2017 the number of birth attended by skilled health personnel worldwide was 78% while in developing countries only 56% of births were attended by skilled health personnel.^1,5^ In Tanzania, births under skilled attendants seems to have taken a step ahead from about 52% of all births being conducted in a health facility and 48% at home in 2010^10^ to 64% of births being conducted in a health facility with a skilled health personnel and 36% of the remaining cases takes place at home.^3^

High incidences of neonatal and maternal morbidity and mortality rates have been displayed amongst unplanned home birth which is not conducted by skilled health personnel.^11^ Postpartum hemorrhage and retained placenta are some of the main adverse outcome of home delivery.^11^ Hypothermia and infections are more associated with neonates who are born at home without the help of skilled health personnel.^12^ Double neonatal mortality rate occur among home births.^13^ Babies born in unplanned home birth without the help of a skilled health attendant are at a higher risk of developing some complications due to hypoxia, infections, respiratory distress, hypothermia acidosis and prematurity.^13^

It is important that mothers deliver their babies in a suitable setting, where there is skilled health personnel, required lifesaving equipment and sterile conditions which can lessen the risk of complications.^14^ Tanzanian government through its National Road Map Strategic Plan to improve Reproductive, Maternal, Newborn, Child & Adolescent Health (2016 to 2020) has set aside resources to increase births assisted by skilled birth attendants.^15^ The aim of the government through its Ministry of Health is for all women to access skilled birth assistance during childbirth. Child survival and good maternal outcome are a result of health facility delivery where an appropriate care from a well-trained team of health personnel is ensured.^13^

A number of studies have worked out factors which influence home childbirth with unskilled assistance to be; low risk perception^16^, low male involvement^17^, poverty^18^, high parity^18^, low knowledge about birth preparedness and complication readiness.^19^

Limited data is available on the factors influencing home childbirth with unskilled birth assistance among women in Dodoma Region. The objective of this study was to determine the factors associated with home childbirth with unskilled birth assistance in Dodoma Region

## METHODS

### Study Design and Setting

**FIGURE 1.**
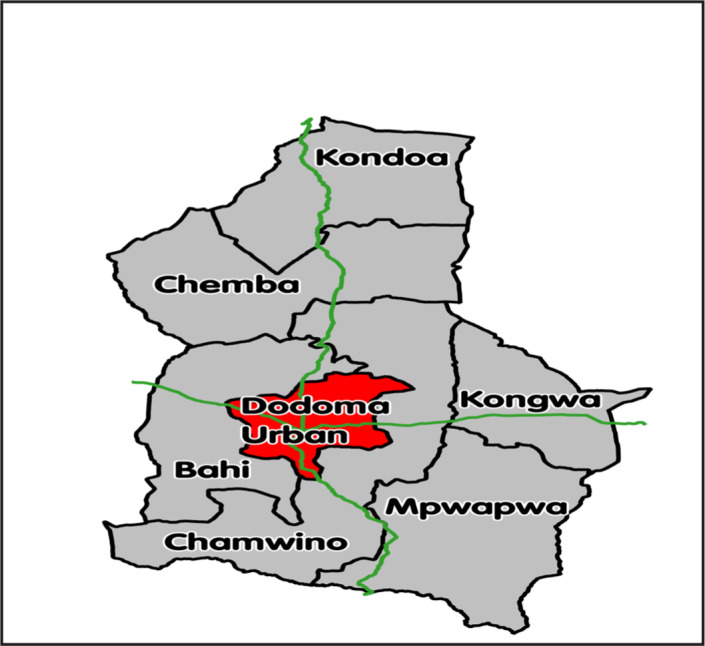
Map of Dodoma Region Showing the Study Area

A descriptive cross sectional study was conducted between March and April 2016 in Dodoma Municipality, Tanzania. Dodoma municipality is subdivided into 4 divisions (Urban, Hombolo, Kikombo and Zuzu Division) comprising of a total of 30 wards and 42 villages/streets. Dodoma Region lies in the eastern-central part of Tanzania and it is the capital city of the country. According to the 2012 national Census, the region had a population of 2,083,588 and population density of 50 people per square kilometres. It covers an area of 41,310 square kilometres (NBS, 2012). Female population accounts for 51.3% of the total population. The annual population growth rate is 2.1% with a sex ratio of 95 males to 100 females [NBS, 2012]. The region's health care service structure comprise of 7 hospitals, 32 health centres, and 269 dispensaries, most of which provide reproductive and child health services^1.9^ The most dominant ethnic group is Gogo but other ethnics exist too such as Sandawe, Rangi Sukuma, Chagga. People of this area are involved in economic activities like business, livestock keeping, office work and farming.^21^ figure 1 below.

### Study Population

The study population was all women of childbearing age who gave birth at the time of data collection and within 3 years prior to data collection.

### Inclusion Criteria

All women of child bearing age who were physically and mentally well, had given birth within 3 years prior to the study, and accepted to sign the consent form for the agreement to participate in a study.

### Exclusion Criteria

Mentally ill mothers plus those that declined to sign the consent form were not included.

### Sample Size

The sample size was calculated using Kish Leslie's formula, n=z^2^p(1-p)/e^2^ where, n is sample size, z=standard normal deviation set at 1.96 at 95%CI, p=prevalence of home delivery in Dodoma region which is 51%^3^ and e= maximum error, assumed to be 5%

n= (1.96)^2^x0.51(1-0.51) from each selected wards=384; 8 wards x 384=3,072 (0.05)^2^

Therefore, the total sample size was 3,072

### Sampling Technique

The three-stage sampling technique was used to obtain the required sample size.

**FIGURE 2.**
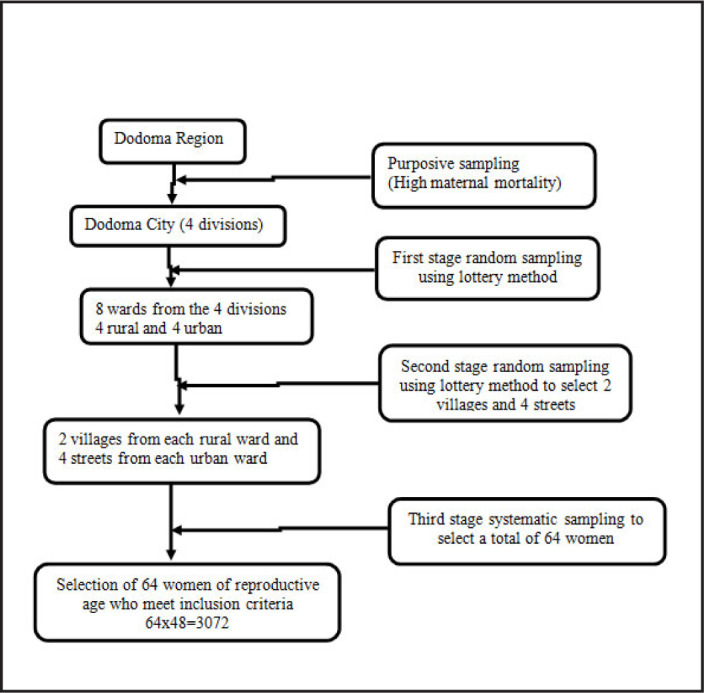
Sampling Technique

In Figure 2, multistage sampling techniques was used to obtain study participants. Dodoma city was selected purposively and all 4 divisions of Dodoma City were included in the study. First stage sampling techniques using simple random sampling (lottery) was used to obtain 8 wards from each division. Second stage sampling using simple random sampling (lottery) was used to obtain 2 villages from each rural ward and 4 streets from each urban ward. The third stage used systematic sampling to select 64 women of reproductive age who met inclusion criteria. In this stage, all hamlets in rural villages were listed and each contributed equal number of women to be included in the study. The first household were selected randomly and were assessed for inclusion criteria. Upon meeting the inclusion criteria and consented to participate, they were included in the study. The next household in a predetermined direction was visited and assessed for inclusion criteria. If the visited household did not meet the inclusion criteria, it was skipped and the followed household was visited until the required number of participants were reached. In the street, similar procedure was used to enrol participants in the study.

### Data Collection

Household survey using structured open and closed ended questionnaire was used. The English version interview questions were translated into Swahili to obtain data from the study participants and to ensure they understand the contents properly. Prior to data collection, the questionnaire was pretested in Madukani Ward, which has almost similar characteristics as the wards selected for study. The questionnaire was modified accordingly before being used in the study. It was administered by 4 research assistants who had recently graduated from medical school and were trained by principal investigator for 3 days before the start of data collection. Questionnaire was given to each eligible woman to fill in. For participants who didn't know how to read and write, face to face interview was conducted by research assistants and the information obtained was filled in by the research assistant

### Data Analysis

The data collected through questionnaires was first coded, and entered into SPSS version 20, owned by IBM corporation company in Chicago city found in The united states of America. Data cleaning was performed before analysing the data so as to identify the incorrect data, missing data during entering and duplicate data. Then data were subjected to simple descriptive statistical analysis. Binary logistic regression model was used to determine association between dependent and independent variable. The variables were entered into bivariate and multivariate logistic regression analysis in order to determine their independent effects in home delivery. The Adjusted ORs and their corresponding 95% Confidence Interval (CI) were obtained. The level of significance was set at P <.05.

### Ethical Consideration

Permission letter to conduct this study was obtained from Research and Ethical Committee of the University of Dodoma. Permission from the local authorities to collect data to the wards and streets was also obtained. The rights of the participant were well protected by obtaining informed consent in oral and written form. Each participant was informed of her right to refuse to participate or not. Confidentiality was assured among the respondent who agreed to participate in the study. The study had ethical clearance number UDOM/DRP/IRRC/14/VOL V/31

## RESULTS

### Socio-demographic Characteristics

A total of 2,523 women were interviewed in this study which make a respond rate of 82%. Majority of interviewed women were aged between 21 and 34 years. 1,588 (62.9%) 1576(62.5%) had primary level education, 1,812 (71.8%) were married, and 1497 (59.3%) had 2 to 4 children Table 1.

**TABLE 1.** The Socio-demographic Characteristics of Interviewed Women (N=2,523)

Variables	Frequency (n)	Percent (%)
**Age group of a participant**		
15-20	328	13
21-34	1,588	62.9
35-49	607	24.1
**Level of Education of a Participant**		
No formal education	514	20.4
Adult education	58	2.3
Primary education	1,576	62.5
Secondary education	330	13.1
Above secondary education	45	1.8
**Tribe of a Participant**		
Gogo	1,628	64.5
Rangi	394	15.6
Others	501	19.9
**Marital Status of a Participant**		
Not married	470	18.6
Married	1,812	71.8
Separated	241	9.6
**Occupation of the Mother**		
House wife	518	20.5
Peasant	1,289	51.1
Pastoralist	134	5.3
Self employed	524	20.8
Employed by the government	48	1.9
Others	10	0.4
**Parity of a Participant**		
1	545	21.6
2-4	1,497	59.3
> 4	481	19.1
**Antenatal Visit**		
Yes	2,370	93.9
No	153	6.1
**Number of Antenatal Visits**		
No antenatal visit	153	6.1
<4 visits	1,445	57.3
>4 visits	925	36.7
**Was the Place of Childbirth Intended?**		
Yes	1,828	72.5
No	695	27.5
**Distance to Nearby Health Facility**		
< 5km	1,373	54.4
> 5km	1,150	45.6

### Prevalence of Home Childbirth among Study Respondents

**FIGURE 3.**
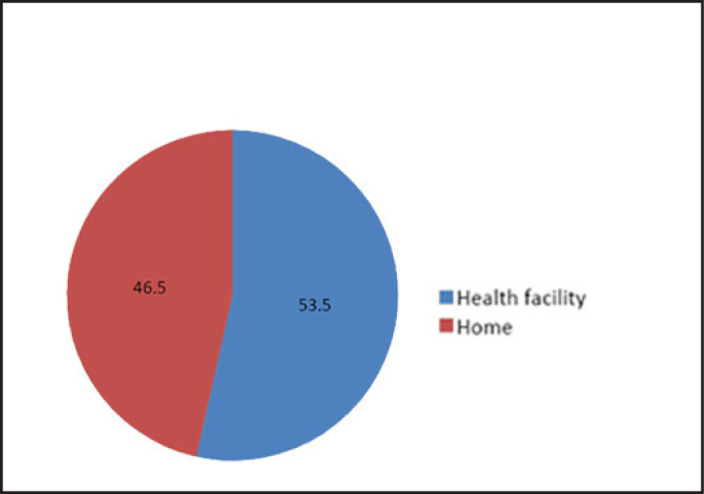
The Prevalence of Home Childbirth in Dodoma

Majority of study participants 1,349 (53.5%) used health facilities for childbirth, 746(29.6%) of deliveries occurred at home assisted by a traditional birth attendant or a relative, 303(12%) of deliveries occurred in traditional birth attendant's home and 125(5%) occurred on the way to a health facility Therefore, the deliveries which occurred in health facilities were 1,349 (53.5%) and those which occurred outside heath facilities were 1,174 (46.5%). In this study, deliveries that occurred outside health facility are termed as home childbirth assisted by unskilled birth attendants (see fig. 3 below).

In table 2 below, all women background characteristics showed a significant relationship with place of childbirth.

**TABLE 2.** The Relationship between Women Background Characteristic and Place of Childbirth (N=2523)

Variables	Health Facility		Home childbirth		X2	p-value
	Frequency (n)	Percent(%)	Frequency (n)	Percent (%)		
**Age Group of a Participant**						
15-20	182	7.2	146	5.8		
21-34	879	34.8	709	28.1	11.65	**
35-49	288	11.4	319	12.6		
**Level of Education of a Participant**						
No formal education	209	8.3	305	12.1		
Adult education	30	1.2	28	1.1		
Primary education	857	34	719	28.5	65.36	***
Secondary education	219	8.7	111	4.4		
Above secondary education	34	1.3	11	0.4		
**Marital Status of a Participant**						
Not married	221	8.8	249	9.9		
Married	1,025	40.6	787	31.2	26	***
Separated	103	4.1	138	5.5		
**Occupation of the Mother**						
House wife	306	12.1	212	8.4		
Peasant	624	24.7	665	26.4		
Pastoralist	89	3.5	45	1.8	36.9	***
Self employed	289	11.5	235	9.3		
Employed by the government	35	1.4	13	0.5		
Others	6	0.2	4	0.2		
**Parity of a Participant**						
1	333	13.2	214	8.5		
2-4	820	32.5	677	26.8	43.42	***
≥4	196	7.8	283	11.2		
**Antenatal visit**						
Yes	1,303	51.7	1067	42.3		
No	46	1.8	107	4.2	35.86	***
**Number of antenatal visits**						
No antenatal visit	46	1.8	107	4.2		
<4	577	22.9	348	13.8	69.24	***
≥4	726	28.8	719	28.5		
**Distance to nearby health facility**						
< 5km	766	30.4	608	24.1		
> 5km	583	23.1	566	22.4	6.005	*

* Indicate significant at p<.05,

** p<.01

*** p<.001.

### Predictors of Use outside Health Facility/Home Childbirth among Study Respondents

After adjusted for the confounders, predictors of home childbirth among study respondents were; level of education [primary education, AOR=0.69 at 95% CI=0.557-0.854, p<.001; secondary education, AOR=0.492 at 95% CI=0.358-0.676, p<.001 and above secondary education, AOR=0.35 at 95% CI=0.16-0.765; p<.01]; marital status [married women, AOR=0.686 at 95% CI=0.547 -0.86, p<.001]; occupation of a mother [peasant, AOR=1.508 at 95% CI=1.214-1.874, p<.05]; After adjusted for the confounders, predictors of home childbirth among study respondents were; level of education [primary education, AOR=0.69 at 95% CI=0.557-0.854, p<.001; secondary education, AOR=0.492 at 95% CI=0.358-0.676, p<.001 and above secondary education, AOR=0.35 at 95% CI=0.16-0.765; p<.01]; marital status [married women, AOR=0.686 at 95% CI=0.547 -0.86, p<.001]; occupation of a mother [peasant, AOR=1.508 at 95% CI=1.214-1.874, p<.05]; parity [2 to 4 children, AOR=1.316 at 95% CI=1.028-1.684, p<.05; More than 4 children, AOR=2.006 at 95% CI=1.427-2.82, p<.001]; number of antenatal visits [4 or more antenatal visits, AOR=0.451 at 95% CI=0.204-0.997, p<.05] and walking distance [less than kilometres, AOR= 0.797 at 95% CI=0.674-0.943, p<.001] (see Table 3)

**TABLE 3.** Bivariate and Multivariate Logistic Regression Analysis to determine the predictors of home childbirth (N=2523)

Variables		95% CI		p-value		95% CI		P-value
	OR	Lower	Upper		AOR	Lower	Upper	
**Age Group of a Participant**								
15-20	1				1			
21-34	1.005	0.792	1.277		0.977	0.728	1.31	
35-49	1.381	1.054	1.808	*	0.877	0.611	1.258	
**Level of Education of a Participant**								
No formal education	1					1		
Adult education	0.64	0.371	1.102		0.776	0.439	1.37	
Primary education	0.575	0.47	0.704	***	0.69	0.557	0.854	***
Secondary education	0.347	0.26	0.463	***	0.492	0.358	0.676	***
Above secondary education	0.222	0.11	0.447	***	0.35	0.16	0.765	**
**Marital Status of a Participant**								
Not married	1				1			
Married	0.681	0.556	0.835	***	0.686	0.547	0.86	***
Separated	1.189	0.87	1.626		0.963	0.684	1.356	
**Occupation of the Mother**								
House wife	1				1			
Peasant	1.538	1.251	1.891	***	1.508	1.214	1.874	***
Pastoralist	0.73	0.49	1.088		0.718	0.474	1.088	
Self employed	1.174	0.918	1.5		1.383	1.066	1.793	*
Employed by the government	0.536	0.277	1.038		1.027	0.491	2.147	
Others	0.962	0.268	3.451		0.793	0.21	2.995	
**Parity of a Participant**								
1	1				1			
2-4	1.285	1.052	1.569	*	1.316	1.028	1.684	*
>4	2.247	1.749	2.886	***	2.006	1.427	2.82	***
**Antenatal Visit**								
No	1				1			
Yes	0.352	0.247	0.502	***	0.608	0.276	1.335	
**Number of Antenatal Visits**								
No antenatal visit	1				1			
<4 visits	0.609	0.515	0.721	***	0.671	0.303	1.483	
≥4 visits	2.349	1.638	3.369	***	0.451	0.204	0.997	*
**Distance to Nearby Health Facility**								
>5km	1				1			
< 5km	0.818	0.699	0.957	*	0.797	0.674	0.943	**

* Indicate significant at p<.05,

** p<.01

*** p<.001.

## DISCUSSION

The current study investigated the prevalence and factors associated with home childbirth. The prevalence of home child birth. The prevalence of home childbirth was found to be 46.5%. This result was higher than the country national coverage of 36%^3^ and higher than what have been observed in the study conducted in Ethiopia in which 6.8% of women gave birth at home.^22^ The possible explanation for the variation in the study findings between these studies could be due to the differences in educational level and variation in socio-economic status of respondents which may have influences in selection of place to give birth. Therefore, awareness about the importance of health facility delivery and the risk of home delivery is needed

The study found that maternal education level has a high influence on choice of place of childbirth. Women with no formal education were more likely to deliver at home compared to women with higher education. Those with primary education were 36% less likely to deliver at home while women with secondary education were 51% less likely to give birth at home. This shows that the more educated the woman is the more likely she would ensure a hospital delivery. This may be because educated mothers have confidence in making their own choices and stick to them and ask for the quality care they desire. Other previous studies conducted in Ethiopia, Kenya and Nepal indicated that the mother's education being lower than primary level is associated with a high prevalence of home delivery.^23–25^ Also most of materials in the antenatal clinics are being provided in writing such as posters. This might be difficult for mothers with no formal education to grasp the material and feel like they are left out, so instead they decide to just be at a where they will be comfortable and not feel like they are left out.^26^

In most of our societies, marital status tends to have an influence on health seeking behaviour. In our study, single mothers were more likely to give birth at home and married women were 32% less likely to give birth at home. A birth process is an amazing experience for almost every woman. It is a moment that no woman can forget and that is why most women will prefer to have the best experience worth remembering. They would want people around to support them through the pregnancy and childbirth. For single mothers this kind of support may not be enough. For married women getting the support from her husband, mother in law and her family may be of help to influence the mother to give birth at the health facility most especially when the husband is the one that makes decision in the family. This is in agreement with the studies that were conducted in Nigeria and Kenya.^23,27^ However, this is in disagreement with the study done in Gambia in which marital status had no influence on a place of delivery for women.^28^

What the mother does for a living can influence her decisions when it comes to pick a place of delivery. This study revealed that peasant women were 1.5% more likely to deliver at home as compared to housewives. Being a peasant can be too demanding for an African mother. Sometimes this can even limit the time that a woman is supposed to focus on her health and her pregnancy. The work may tend to take a greater part of her life and with this the woman may not have a chance to access the information on the importance of giving birth at the health facility and hence she may not see the importance of it at all. This is in line with previous studies that showed occupation of the mother can influence home delivery.^29^

Giving birth to most of the mothers is all about experience. The attitude of a prime gravida may not be the same as that of the multipara. This study can vividly prove that women with parity of 2 to 4 were 1.3% more likely to deliver at home while those of more than 4 children were 2 times more likely to give birth at home. If a woman gave birth more than 4 times without any complications, she may see it as just a normal process hoping that everything will just go smoothly as other pregnancies and there is nothing to worry about. This is even more likely to happen to those women who had their last birth at home. The woman feels like she has enough experience to handle all that without the help of medical personnel. The other reason can be that the responsibility of taking care of the children is put on the hands of the mother, with this in mind the mother of higher parity will be concerned with who will take care of the family once she is gone and this can affect her decision. This is in agreement with other studies which were conducted in Ethiopia and Tanzania.^30,31^

Antenatal visit plays a vital role when it comes for a woman to pick a place of delivery. There is a lot of helpful health information that women get from these ANC visits. This study shows that women with less than 4 antenatal visits were 40% less likely to deliver at home compared to women who have never attended any antenatal care. Also, women with more than 4 antenatal visits were 2 times more likely to give birth at a health facility compared to women who never attend antenatal clinic. Women with more antenatal visits are less likely to give birth at home possibly because of the content of health education and understanding the risk factors that may accompany home delivery. This is in line with studies conducted in other countries indicating that more antenatal care visits decrease the likelihood of giving birth at home.^32^ The findings from this study is in contrary with the findings reported in Ethiopia.^22^

In most of developing countries, there is no enough health facilities. Even the few that are there may be too far for some women to access them. This has been a major setback in increasing facilities delivery. In this study women who lived less than 5km from the health facility were 21% less likely to give birth at home compared to women who lived more than 5km from the health facility. This can be due to variety of reasons including the fact that some women cannot afford the transport fare to reach the health centre. Also, due to lack of birth preparedness knowledge, most women do not have a transport plan in hand in case of sudden labour. Another reason is, the physical characteristic of a particular place which makes it hard to make it to the nearby health facility on time even when the transport is accessible. Some women also feel burdened to leave their family behind and go far to seek health care. Women may also feel like they are disturbing their relatives who will have to travel long distances to check them up in the health care facility. This is in line with other previous studies which indicated that distance to the health facility has an association with home delivery.^28,29^

The strength of this study is the use of large sample size which allow researchers to better determine the average values of prevalence of home childbirth in Dodoma Region. Also, the study used quantitative study approach which allow the generalisation of the findings. Nevertheless, this study is not without limitations. It was a cross-sectional study which relied on participants recalling the past event which could introduced recall bias. The limitation was minimised by including women who gave birth during the time of data collection and who had given birth in the period of 3years prior the time of data collection.

## CONCLUSION

The number of women who are not using health facilities for childbirth is still high thus requiring prompt intervention. The predictors of using health facilities for childbirth were; the level of education of pregnant women, marital status, parity where the more the number of children a woman has the less likely she will use the health facility for childbirth, number of antenatal visits where those with few visits are less likely to use health facility for childbirth and distance of Health Facility where women living far from health facilities are less likely to use health facilities for childbirth. More innovative interventions are needed to increase the use of health facilities for childbirth in this central part of Tanzania.
